# Hsp90 Binds Directly to Fibronectin (FN) and Inhibition Reduces the Extracellular Fibronectin Matrix in Breast Cancer Cells

**DOI:** 10.1371/journal.pone.0086842

**Published:** 2014-01-22

**Authors:** Morgan C. Hunter, Kyle L. O’Hagan, Amy Kenyon, Karim C. H. Dhanani, Earl Prinsloo, Adrienne L. Edkins

**Affiliations:** Biomedical Biotechnology Research Unit, Department of Biochemistry, Microbiology and Biotechnology, Rhodes University, Grahamstown, Eastern Cape, South Africa; University of Pittsburgh, United States of America

## Abstract

Heat shock protein 90 (Hsp90) has been identified in the extracellular space and has been shown to chaperone a finite number of extracellular proteins involved in cell migration and invasion. We used chemical cross-linking and immunoprecipitation followed by tandem mass spectrometry (MS/MS) to isolate a complex containing Hsp90 and the matrix protein fibronectin (FN) from breast cancer cells. Further analysis showed direct binding of Hsp90 to FN using an *in vitro* co-immunoprecipitation assay, a solid phase binding assay and surface plasmon resonance (SPR) spectroscopy. Confocal microscopy showed regions of co-localisation of Hsp90 and FN in breast cancer cell lines. Exogenous Hsp90β was shown to increase the formation of extracellular FN matrix in the Hs578T cell line, whilst knockdown or inhibition of Hsp90 led to a reduction in the levels of both soluble and insoluble FN and could be partially rescued by addition of exogenous Hsp90β. Treatment of cells with novobiocin led to internalization of FN into vesicles that were positive for the presence of the lysosomal marker, LAMP-1. Taken together, the direct interaction between FN and Hsp90, as well as the decreased levels of both soluble and insoluble FN upon Hsp90 inhibition or knockdown, suggested that FN may be a new client protein for Hsp90 and that Hsp90 was involved in FN matrix assembly and/or stability. The identification of FN as a putative client protein of Hsp90 suggests a role for Hsp90 in FN matrix stability, which is important for a number of fundamental cellular processes including embryogenesis, wound healing, cell migration and metastasis.

## Introduction

Heat shock protein 90 (Hsp90) is one of the most abundant and ubiquitously expressed chaperone proteins, constituting approximately 1–2% of the total cell protein complement [1], [2], [3], [4]. Playing a vital role in cell survival, Hsp90 modulates the stability and transport of a diverse set of more than 200 client proteins [5]. Hsp90 does not function alone, but rather forms part of a multichaperone complex with other chaperones, co-chaperones and client proteins that work synergistically in the folding, maturation, transport, activation and inactivation of proteins involved in vital cellular processes [3], [6]. Hsp90 plays a fundamental role in the stability, refolding and maturation of key oncogenic proteins expressed in tumour cells [5], [7]. These oncogenic client proteins include numerous transcription factors (E.g. Signal Transducer and Activator of Transcription 3 [STAT-3]), cell receptors (E.g. Epidermal Growth Factor Receptor [EGFR]) and signalling kinases (E.g. Protein Kinase B [PKB]) [4], [5], [8], [9], [10], [11], [12] [13]. A comprehensive and regularly updated list of Hsp90 client proteins can be found on the Picard website (http://www.picard.ch/downloads/Hsp90interactors.pdf).

Five human isoforms of Hsp90 have been identified. The cytosolic isoforms comprise the inducible Hsp90α and the constitutively active Hsp90β [14]. Organelle Hsp90 isoforms include the ER isoform, (glucose-regulated protein; Grp94/Gp96), mitochondrial TNF receptor-associated protein 1 (Trap1), and Hsp90N that shares a high sequence homology with Hsp90α and Hsp90β but lacks the 25 kDa N-terminus [15]. It is thought that Hsp90N is primarily membrane associated due to its unique hydrophobic N-terminal domain [16]. Recent evidence showed that both Hsp90α and Hsp90β have been identified in the extracellular space of many cancers [17], [18], [19]. Export of Hsp90 does not occur via the canonical signal sequence pathway; it is thought to be secreted via a non-classical trafficking pathway [20]. Recent evidence shows the secretion of extracellular Hsp90α via exosomes in immune and other physiologically normal cell types [20]. Given that Hsp90 has been detected on the cell surface of numerous cells [2], [12], [18], [19], as well as in the extracellular space [21], [22], [23], it is reasonable to hypothesize that Hsp90 resides both as a pool of extracellular soluble Hsp90, as well as a pool of membrane associated Hsp90. Some studies have demonstrated that both Hsp90α and Hsp90β are secreted as soluble Hsp90 [24], while others showed the presence of Hsp90α on the surface of cancer cells coupled with secretion of Hsp90α, and not Hsp90β, into the extracellular space [18], [21], [22]. Extracellular Hsp90 has been shown to chaperone a finite number of extracellular proteins predominantly involved in cell migration and invasion [18]. These clients include matrix metalloproteinase-2 (MMP-2) [18], matrix metalloproteinase-9 (MMP-9) [25], low density lipoprotein receptor-related protein 1 (LRP-1/CD91) [26], epidermal growth factor receptor 2 (HER-2) [12], and tissue plasminogen activator (TPA) [20], [27]. There is evidence that Hsp90 may chaperone both extracellular soluble and extracellular membrane-associated proteins [28], [29].

We report that the ECM protein, fibronectin (FN), bound directly to Hsp90 *in vitro* and could be isolated in a complex with Hsp90 from breast cancer cells. Exogenous Hsp90β was shown to increase the formation of extracellular FN matrix and inhibition or knockdown of Hsp90 isoforms led to a reduction in the levels of extracellular FN matrix. Inhibition of Hsp90 with novobiocin was shown to result in FN internalization through an endocytosis mediated process that could be inhibited by treatment of cells with methyl-β-cyclodextran. Taken together, these data suggested that FN may be a client of Hsp90. The function of FN is multifaceted and includes the regulation of embryogenesis, mesoderm formation, tissue repair, cell migration, differentiation, cell growth as well as certain pathological disorders such as fibrosis, tumour development and atherosclerosis [30], [31], [32], [33], [34]. The identification of FN as a putative client protein of Hsp90 may be one mechanism by which Hsp90 regulates cell migration and invasion. Given the role of Hsp90 in promoting cancer cell migration and invasion, a critical contributor to metastatic progression, it is reasonable to hypothesise that client proteins of Hsp90 may be coupled with degradation and remodelling of the extracellular matrix (ECM).

## Methods

### Cell Culture

The Hs578T (HTB-126), MCF-7 (HTB-22) and MDA-MB-231 (HTB-26) breast cancer cell lines were obtained from the American Type Culture Collection (ATCC). The primary human fibroblast (FG) cell line was a generous gift from Dr Sharon Prince (University of Cape Town, South Africa) and has been described previously [35]. MDA-MB-231 and MCF-7 breast cancer cells were maintained in Dulbecco’s Modified Eagle Medium (DMEM) supplemented with GlutaMAX™-I (Gibco, Invitrogen, UK), 5% (v/v) fetal calf serum (FCS; Gibco, Invitrogen, UK) and penicillin/streptomycin (100 U.ml^−1^; Biowhittaker, UK). Hs578T breast cancer cells were maintained in DMEM supplemented with GlutaMAX™-I, 10% (v/v) FCS, penicillin/streptomycin (100 U.ml^−1^) and 2 mM insulin (Novorapid, Canada). FG cells were maintained in DMEM supplemented with GlutaMAX™-I, 10% (v/v) FCS, penicillin/streptomycin (100 U.ml^−1^). All cells were maintained at 37°C in a humidified atmosphere with 10% CO_2_.

### Cell Surface Biotinylation and Streptavidin-agarose Affinity Chromatography

Confluent MDA-MB-231 cells were biotinylated by incubation with NHS-biotin (1 mg.ml^−1^) for 30 minutes at 4°C. Cells were quenched with 1 M Tris-HCl (pH 7.5) and washed twice with PBS (137 mM NaCl, 2.7 mM KCl, 10 mM Na_2_PO_4_, 2 mM KH_2_O_4_) to remove unbound NHS-biotin. Cells were subsequently lysed in RIPA buffer (50 mM Tris-HCl, pH7.4, 150 mM NaCl, 1 mM ethyleneglycol-bis[beta-aminoethylether] N’N’N’N-tetraacetic acid/ethylenediaminetetraacetic acid [EGTA/EDTA], 1 mM Na_3_VO_4_, 1% [v/v] NP40, 1 mM sodium deoxycholate [DOC], 1 mM phenylmethylsulfonyl fluoride [PMSF] and 0.05% [v/v] protease inhibitor cocktail) after being lifted either by scraping or by trypsin treatment. Cell supernatants were incubated with streptavidin-agarose for 1 hour at 4°C and bound protein harvested by centrifugation at 13 000 rpm in a microfuge for 1 minute at 4°C. Purified proteins were released in SDS-PAGE sample buffer (60 mM Tris-HCl, pH 6.8, 2% [w/v] SDS, 10% [v/v] glycerol, 0.01% [w/v] bromophenol blue, 5% [v/v] β-mercaptoethanol) by boiling for 8 minutes. Purified proteins were analyzed according to the SDS-PAGE and immunoblotting analysis methodology using mouse anti human Hsp90α/β (sc-13119, Santa Cruz Biotechnology, USA), rat monoclonal anti human Hsp90α (ADI-SPA-840, Enzo Life Sciences) and mouse anti human Hsp90β (ab119833, Abcam, UK) for the detection of total Hsp90 and specific Hsp90α and Hsp90β isoforms respectively.

### Chemical Crosslinking of Cell Surface Proteins

Surface proteins of MDA-MB-231 cells were chemically crosslinked with either the cell impermeable bissulfosuccinimidyl suberate (BS3) crosslinker (5 mM; 21580, Thermo Scientific, USA) or the thiol-cleavable, cell impermeable 3,3′–dithiobis(sulfosuccinimidyl)propionate (DTSSP) crosslinker (2 mM; 21578, Thermo Scientific, USA) for 1 hour at 4°C followed by quenching with 20 mM Tris-HCl (pH 7.5). Cell lysates were immunoprecipitated with mouse anti human Hsp90α/β primary antibody or BSA (negative control) using the Dynabeads® Co-immunoprecipitation kit (Invitrogen, UK) according to the manufacturer’s instructions. Cleavage of the crosslinker was performed with 5% (v/v) β-mercaptoethanol in SDS-PAGE sample buffer. Immunoprecipitated proteins were analyzed as per the SDS-PAGE and immunoblotting analysis methodology using mouse anti human Hsp90α/β for the detection of Hsp90.

### SDS-PAGE and Immunoblotting Analysis

Proteins were separated by discontinuous SDS-PAGE using the Laemmli buffer system [36]. Protein concentrations were determined using the NanoDrop 2000™ (Thermo Scientific) and equal amounts of total protein loaded per well. Resolved proteins were transferred onto nitrocellulose membrane using transfer buffer (25 mM Tris, 192 mM glycine, 20% [v/v] methanol), at 100 V for 2 hours on ice with stirring [37]. Each membrane was blocked in blocking solution (5% [w/v] non-fat milk powder in TBS [50 mM Tris, 150 mM NaCl]) for 1 hour. Membranes were incubated with primary antibody at 4°C overnight with shaking. Membranes were subsequently washed with TBST (TBS containing 1% [v/v] Tween 20). Fresh blocking solution was added with the addition of HRP-linked species specific secondary antibodies. Incubation with species matched secondary antibodies was performed for 1 hour at room temperature, after which the membranes were washed with TBST. Proteins were detected using the ECL Advance Western Blotting Detection Kit (Amersham, UK) and visualized using the Chemidoc™ system (Biorad).

### Tandem Mass Spectrometry

Crosslinked Hsp90 protein complexes isolated by immunoprecipitation were resolved on an 8% non-reducing SDS-PAGE gel and detected by colloidal Coomassie staining [38]. Protein bands were excised from the gel and subjected to trypsin digestion [39]. Digested peptides were concentrated on a C18 reverse phase trapping column, followed by separation on a custom packed 15 cm C18 reverse phase column. Peptides were eluted off the column at 350 nl.min^−1^ using an Agilent Nano HPLC with a 10–25% (v/v) Acetonitrile/0.1% (v/v) formic acid gradient. Eluted peptides were analyzed on an AB SCIEX 4000QTRAP hybrid triple quadrupole ion trap mass spectrometer with a nanospray source. A survey scan was performed for eluting peptides between 400 and 1200 Daltons, followed by an enhanced resolution scan to determine the charge state of each peptide before fragmenting the peptides in the collision cell. Peptide sequence information was analyzed by an in-house Mascot (Matrix Science) server using the SwissProt database (SwissProt_DB 11082010), with results limited to Homo sapiens (20359 sequences). Queries identifying keratin were regarded as contaminants and removed from the protein hits list. Non-significant results were removed from the results output.

### *In vitro* Co-immunoprecipitation Assay

An *in vitro* co-immunoprecipitation assay was performed using the Dynabeads® co-immunoprecipitation kit. Mouse anti human Hsp90α/β primary antibody (50 µg) or BSA (negative control) was covalently coupled to magnetic Dynabeads® (5 mg), following the manufacturer’s instructions. Hsp90β antibody or BSA bound beads were incubated with 1 µg Hsp90β (SPR-102A/B/C, StressMarq, USA) and/or soluble FN (sc-29011, Santa Cruz Biotechnology, USA) for 15 minutes, washed and immunoprecipitates eluted. Hsp90β (1 µg) and FN (1 µg) were loaded as input controls. Co-immunoprecipitated proteins were resolved by SDS-PAGE and analyzed by immunoblotting and silver staining using the PageSilver™ Silver Staining Kit (K0681, Fermentas, USA).

### Solid Phase Binding Assay

FN (100 µg.ml^−1^) in 50 µl buffer A (20 mM Tris-HCl, 150 mM NaCl, pH 7.4, 1 mM ATP, 5 mM CaCl_2_, 0.05% [v/v] Tween 20) was incubated in the wells of a flat-bottomed high binding 96-well microplate (655061, Greiner Bio-One, UK) for 5 hours at 22°C. Non-specific binding sites were blocked with 3% (w/v) BSA in buffer A. Blocking solution was removed and either GST (negative control) or GST-Hsp90β (0–2500 nM) incubated in each well for 12 hours at 4°C. Wells were subsequently washed with 1% (w/v) BSA in buffer A and incubated with goat anti schistosomal GST primary antibody (27457701, GE Healthcare, UK) in buffer A (1∶1000) for 2 hours at 22°C. Wells were washed, followed by the addition of donkey anti goat IgG-HRP secondary antibody (sc-2020, Santa Cruz Biotechnology, USA) in buffer A (1∶4000) for 1 hour at 22°C. Tetramethylbenzidine (TMB) substrate solution (in 0.05 M Phosphate-Citrate, pH 5.0) was added to each well for 25 minutes, and the reaction was stopped with 2M H_2_SO_4_. The absorbance at 450 nm was recorded and data analysis performed using GraphPad Prism 4.03 (Graphpad Software, Inc., USA). FN adsorption to the ELISA plate was confirmed with mouse anti human fibronectin primary antibody (F0916, Sigma Aldrich, USA) using a similar protocol.

### Surface Plasmon Resonance (SPR)

Surface plasmon resonance (SPR) spectroscopy was performed on a ProteOn™ XPR36 Protein Interaction Array System (Bio-Rad, US) at 25°C using running buffer (40 mM HEPES-NaOH, pH 7.4, 150 mM KCl, 5 mM MgCl_2_). A ProteOn™ GLC Sensor chip (#176-5011, Bio-Rad, US) was initialised with 50% (v/v) glycerol and preconditioned at 30 µl.min^−1^ with successive 60 µl pulses of 0.05% SDS (w/v) and 100 mM HCl in both the horizontal and vertical direction. Activation of the GLC chip surface followed with a 150 µl pulse of a 1∶1 mixture of 1-ethyl-3-(3-dimethylaminopropyl)carbodiimide (EDAC) and Sulfo-NHS at 30 µl.min^−1^. Recombinant Hsp90α or Hsp90β (10 µg.ml^−1^), in 10 mM sodium acetate, pH 4.5 (determined by preconcentration pH scouting), was immobilized. Free amines on a third ligand channel were blocked with 1 M ethanolamine and used as an inline reference. FN sensorgrams were collected at a 100 µl.min^−1^ injection for 120 seconds followed by a 600 second delay used to monitor dissociation. Regeneration was performed with an 18 second pulse injection of 10 mM Tris, pH 8.0, 3 M guanidine-HCl. Blank buffer injections were used as a double reference subtraction. Further analysis was conducted in GraphPad Prism 4.03. SPR supplementary data was acquired as outlined in supplementary figure legends.

### Confocal Microscopy

Cells were seeded at a density of 4.5×10^5^ cells.ml^−1^ onto sterile glass cover slips and allowed to adhere overnight followed by treatments indicated in figure legends. Cells on coverslips were fixed with ethanol and blocked with 1% (w/v) BSA/PBS. Fixed cells were incubated with primary antibody in 1% (w/v) BSA/PBS (1∶100), washed with 1% (w/v) BSA/PBS, and subsequently incubated with species specific fluorescently tagged secondary antibody in 1% (w/v) BSA/PBS (1∶500). Antibodies used are specified in figure legends. Nuclei were stained with Hoechst 33342 (1 µg.ml^−1^). Images were captured using the Zeiss LSM 510 Meta laser scanning confocal microscope and analyzed using Zen software (blue edition, Zeiss, Germany) or AxiovisionLE 1.4.7 (Carl Zeiss Imaging Solutions Germany). Product of the Differences from the Mean (PDM) values and a Colour Scatter Plot were generated using Image J 1.43m. Where indicated, the degree of extracellular FN staining was quantified using ImageJ (NIH, USA) and the average mean gray value of at least three separate images performed in triplicate is reported. Antibodies used in fluorescent staining were mouse anti-human FN (F0916) and rabbit anti human FN (F3648) from Sigma Aldrich, USA. Goat anti human Hsp90α/β (sc-1055) and mouse anti LAMP1 (sc17768) were from Santa Cruz Biotechnology, USA. Donkey anti mouse Dylight® 488, donkey anti rabbit Dylight® 555 and donkey anti goat DyLight® 650 were from Pierce while donkey anti mouse Alexa fluor 488, donkey anti rabbit Alexa Flour 550 were from Invitrogen, USA.

### Transient Knockdown of Hsp90 by Short Interfering RNA

Hs578T cells, serum starved in antibiotic-free media (DMEM with 2 mM insulin) for 8 hours, were transiently transfected using siRNA oligonucleotides (25 nM) in Dharmafect 1 transfection reagent (Dharmacon, USA) for 48 hours. Knockdown of Hsp90α or Hsp90β was performed using isoform specific siRNA (Hsp90α: M-005187-02, Hsp90β: M-005186-02, Dharmacon, USA) or a scrambled, non-targeting pool of siRNA oligonucleotides (D-001206-13-05, Dharmacon, USA) to serve as a negative control for any observed knockdown. Levels of Hsp90 knockdown were analyzed by immunoblotting using mouse anti human Hsp90α (SMC-147A/B, StressMarq, USA) and mouse anti human Hsp90β (SMC-107A/B, StressMarq, USA) primary antibodies. To analyse the effect of Hsp90 knockdown on soluble and insoluble FN, siRNA transfected cells were cultured for an additional 24 hours in complete growth medium and processed for confocal microscopy or as per the deoxycholate (DOC) assay.

### Deoxycholate (DOC) Assay for Fractionation of Soluble and Insoluble FN

The DOC assay was performed as outlined by Brenner and colleagues [40]. Hs578T cells were seeded in a 6-well culture dish at a density of 3×10^5^ cells.ml^−1^ and incubated overnight. Fresh media was added the following day and cells were harvested by scraping into DOC lysis buffer (2% [w/v] deoxycholate, 20 mM Tris-HCl, pH 8.8, 2 mM PMSF, 2 mM EDTA and 0.05% [v/v] protease inhibitor cocktail). Soluble and insoluble fractions were separated by centrifugation at 13 000 rpm in a microfuge for 20 minutes. The supernatant (soluble fraction) was removed and cell pellet resuspended in SDS buffer (1% [w/v] SDS, 25 mM Tris-HCl, pH 8.0, 2 mM PMSF, 2 mM EDTA and 0.05% [v/v] protease inhibitor cocktail). Protein concentrations were determined using a NanoDrop 2000™. Levels of soluble and insoluble fractions were determined by immunoblotting using mouse anti human FN primary antibody. Levels of GAPDH and/or Histone H3 were determined using rabbit anti human GAPDH (sc-25778, Santa Cruz Biotechnology, USA) and rabbit anti human Histone H3 (#9715, Cell Signalling Technology®, USA) and used to ensure equal protein loading.

### Fluorescent Labeling of Purified FN

Lyophilized FN (1 mg) was reconstituted in a borate buffer (0.67 M) to a final concentration of 2 mg.ml^−1^. The total volume of FN was transferred to a vial of DyLight® 550 reagent (62263, Thermo Scientific, USA), vortexed and incubated for 1 hour at room temperature in the dark. Excess DyLight® reagent was removed by centrifugation through a Zeba spin desalting column (89882, Thermo Scientific, USA) at 1000×*g* for 1 minute. The concentration of labeled FN as well as the moles of dye per mole of protein was determined using the following calculations: Protein concentration (M) = [A_280_–(A_max_×CF)]÷ε_protein_×dilution factor. Moles dye per mole protein = (A_max_ of the labeled protein×dilution factor)÷(ε_dye_×protein concentration [M]). For CFSE studies, lyophilized FN (1 mg) was reconstituted in 0.1 M sodium bicarbonate buffer to reach a final concentration of 2 mg.ml^−1^. CFSE (10 mg.ml^−1^, 08951, Sigma Aldrich, USA), solubilised in DMSO was added to the dissolved FN with gentle agitation. The reaction was incubated for 1 hour at room temperature with continuous agitation followed by the addition of stop reagent (1.5 M hydroxylamine, pH 8.5) for 1 hour at room temperature. Unbound CSFE reagent was removed from labelled FN through a Zeba spin desalting column by centrifugation at 1000×*g* for 1 minute. Protein concentration was determined at 280 nm and equal amounts of total FN-CFSE were added to samples as indicated in the figure legends. For flow cytometric analysis, cells were lifted with trypsin and proteinase K treatment and resuspended to 5×10^6^ cells.ml^−1^ in PBS. FN-CFSE fluorescence was detected by excitation at 488 nm and emission collection using the FITC channel on a FACSAria II flow cytometer. A total of 20 000 live events were captured for all analyses. Confocal analysis was performed as described previously.

### Live Cell Imaging of Fluorescent FN Matrix

Cells were seeded into sterile glass-bottomed cell culture dishes and allowed to adhere overnight at 37°C in normal medium supplemented with 50 nM DyLight® 550-labeled FN. The following day the medium was removed and replaced with normal medium lacking phenol red. The Zeiss LSM 510 Meta laser scanning confocal microscope was used for live cell imaging. Prior to treating cells, the microscope unit and stage was pre-warmed to 37°C and a regulated concentration of 10% CO_2_ was maintained. Cells were either untreated or treated with novobiocin (1 mM) and mounted onto the microscope stage using the 63×oil objective. A time series experiment was prepared such that 36 images were captured at 15 minute intervals, with a total incubation period of 9 hours. Images were analysed using AxiovisionLE 4.7.1 software.

## Results

### Hsp90 Existed as Part of a Higher Molecular Weight Complex on the Surface of MDA-MB-231 Cells

**Figure 1:**
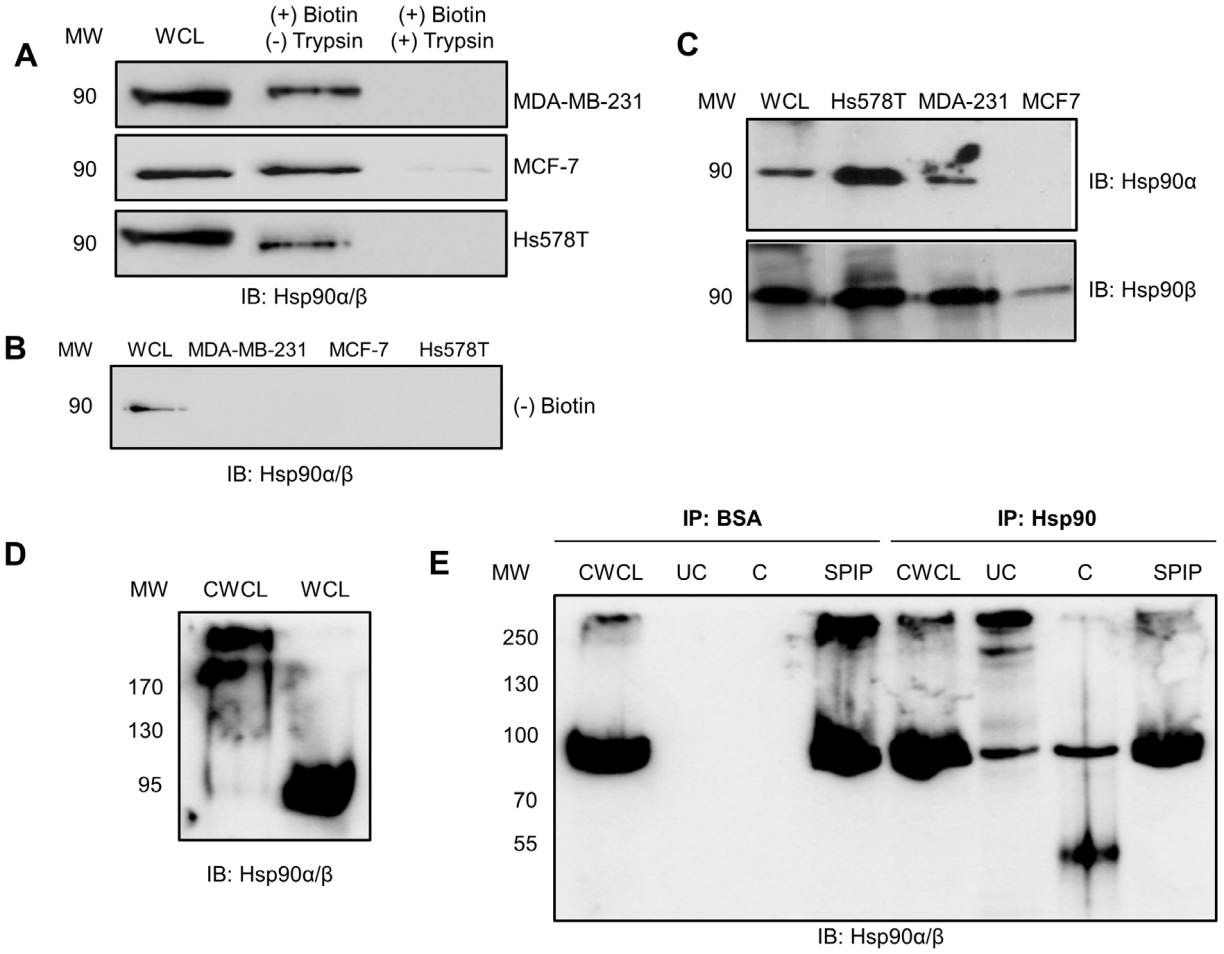
Hsp90 is present in a putative complex on the plasma membrane of MDA-MB-231 cells. (**A**) Confluent MDA-MB-231, MCF-7 and Hs578T cells were subject to (+) biotin and (±) trypsin treatment. Biotinylated proteins were purified and equal amounts of protein resolved by SDS-PAGE and probed for Hsp90α/β by immunoblotting. Whole cell lysates (WCL) are shown as a reference. (**B**) MDA-MB-231, MCF-7 and Hs578T cells treated without NHS-biotin were probed for Hsp90α/β by immunoblotting and showed no Hsp90 in the purified fraction. WCL of Hs578T cells were used as a positive control for immunoblotting. (**C**) Biotinylated fractions from equal numbers of Hs578T, MDA-MB-231 or MCF-7 cells were probed for the presence of either Hsp90α or Hsp90β using isoform specific antibodies. The WCL from Hs578T cells was used as a positive control for immunoblotting. (**D**) Surface proteins of MDA-MB-231 cells were chemically crosslinked with BS3 reagent and the crosslinked whole cell lysate (CWCL) and whole cell lysate (WCL) probed for Hsp90α/β by immunoblotting. (**E**) Surface proteins of MDA-MB-231 cells were chemically crosslinked with the thiol-cleavable DTSSP reagent and cell lysates immunoprecipitated with either BSA (IP: BSA; Negative control) or Hsp90α/β primary antibody (IP: Hsp90). The crosslinked whole cell lysate (CWCL), uncleaved (UC) and cleaved (C) immunoprecipitates, and supernatant post immunoprecipitation (SPIP) were probed for Hsp90α/β by immunoblotting. The data shown are representative of results obtained from triplicate experiments in all cases.

In order to validate the presence of extracellular Hsp90 in our cell lines, the surface proteins of the breast cancer cell lines MDA-MB-231, MCF-7 and Hs578T were labelled by biotin tagging (using cell impermeable NHS-biotin) and isolated using biotin-streptavidin affinity purification. Isolated fractions were resolved by SDS-PAGE and probed for the presence of Hsp90α/β by immunoblot analysis (Figure 1). Biotinylation was performed for 30 minutes at 4°C to prevent internalisation of NHS-biotin by the cell, and to ensure that only extracellular species were affinity purified [41]. The immunoblot for Hsp90α/β revealed that Hsp90 was observed in the affinity purified fraction of all three cell lines (Figure 1A). Cells treated with trypsin after biotinylation showed a loss of Hsp90 in the biotin purified fraction, indicating that cleavage of the extracellular proteins by trypsin resulted in loss of Hsp90. Cells treated without NHS-biotin showed no Hsp90 in the purified fraction (Figure 1B). These data supported the conclusion that Hsp90 in the biotinylated fraction was extracellular. The levels of Hsp90 isolated in the biotinylated fractions consistently appeared lower than those of the whole cell lysate (WCL), suggesting only a portion of the total Hsp90 was extracellular. Immunoblotting of the biotinylated fractions from (A) was performed using isoform specific antibodies. Both Hsp90α and Hsp90β were detected in the biotinylated fractions of Hs578T and MDA-MB-231 cell lines, while Hsp90β and not Hsp90α was detected in biotinylated fractions from MCF-7 cells (Figure 1C). Our observations of extracellular Hsp90α and Hsp90β isoforms in breast cancer cell lines are supported by previous reports of other researchers [2], [4], [15], [18], [21], [22], [23]. Immunostaining of live cerebellar cells showed Hsp90α and Hsp90β isoforms localized on the cell surface [42]; Hsp90β has also been reported on the cell surface of cultured oligodendrocyte precursor cells (OPCs) by peptide mass fingerprinting [43], while Hsp90α was found in conditioned medium of HT-1080 cells [11], human dermal fibroblasts [21] and vascular smooth muscle cells [22].

To determine whether extracellular Hsp90 was present in a complex with any additional proteins, cell surface proteins of MDA-MB-231 cells were crosslinked using the cell impermeable, non-cleavable crosslinker BS3 and probed for Hsp90α/β (Figure 1D). The cell impermeable chemical crosslinker used, BS3, crosslinked all proteins within 11.4 Å of each other [44]. Treatment of MDA-MB-231 cells with BS3 led to the presence of higher molecular weight species (>200 kDa) of Hsp90α/β in the crosslinked whole cell lysate (CWCL) compared to the untreated whole cell lysate (WCL). Although the higher molecular weight species were insufficiently resolved, more than one prominent band was visible; suggesting the presence of more than one putative complex containing Hsp90. Uncomplexed Hsp90 was still present in the CWCL as seen by the presence of a band at 90 kDa. MDA-MB-231 cells treated without the crosslinker still showed the presence of Hsp90 as a definitive band with size 90 kDa in the WCL. These data suggested the presence of a complex containing extracellular Hsp90 that was membrane associated, or soluble but closely associated with the plasma membrane.

We subsequently developed an immunoprecipitation technique to isolate Hsp90 containing crosslinked complexes (Figure 1E). Crosslinked complexes were immunoprecipitated from DTSSP treated whole cell lysates of MDA-MB-231 cells using a mouse monoclonal antibody against Hsp90α/β (IP: Hsp90). DTSSP is the cleavable crosslinker equivalent to BS3 and contains a disulphide bond that can be cleaved using reducing agents [45]. Beads covalently linked to BSA served as a control for any non-specific binding of protein to the beads during immunoprecipitation (IP: BSA). Immunoprecipitated proteins were treated with SDS loading buffer with (cleaved) or without (uncleaved) 5% (v/v) β-mercaptoethanol and the presence of Hsp90 determined by immunoblotting. Hsp90 was not detected in either the uncleaved (UC) or cleaved (C) fractions from the control immunoprecipitation using BSA-conjugated beads (IP: BSA). Non-crosslinked Hsp90 (90 kDa) as well as distinct higher molecular weight species of Hsp90 (>200 kDa) were detected in the cross-linked whole cell lysate (CWCL) as well as the uncleaved (UC) fraction after immunoprecipitation using anti-Hsp90α/β antibody (IP: Hsp90). The higher molecular weight species containing Hsp90 were lost upon cleavage of the DTSSP crosslinker with β-mercaptoethanol. An expected increase in the intensity of Hsp90 at 90 kDa was observed, as well as a lower molecular weight band at 45 kDa, which we predict to be a result of cleavage of Hsp90. A similar sized Hsp90 cleavage product has been observed upon treatment with deoxycholate [46].

### Isolation of an Extracellular Hsp90 Complex Containing Fibronectin

**Figure 2:**
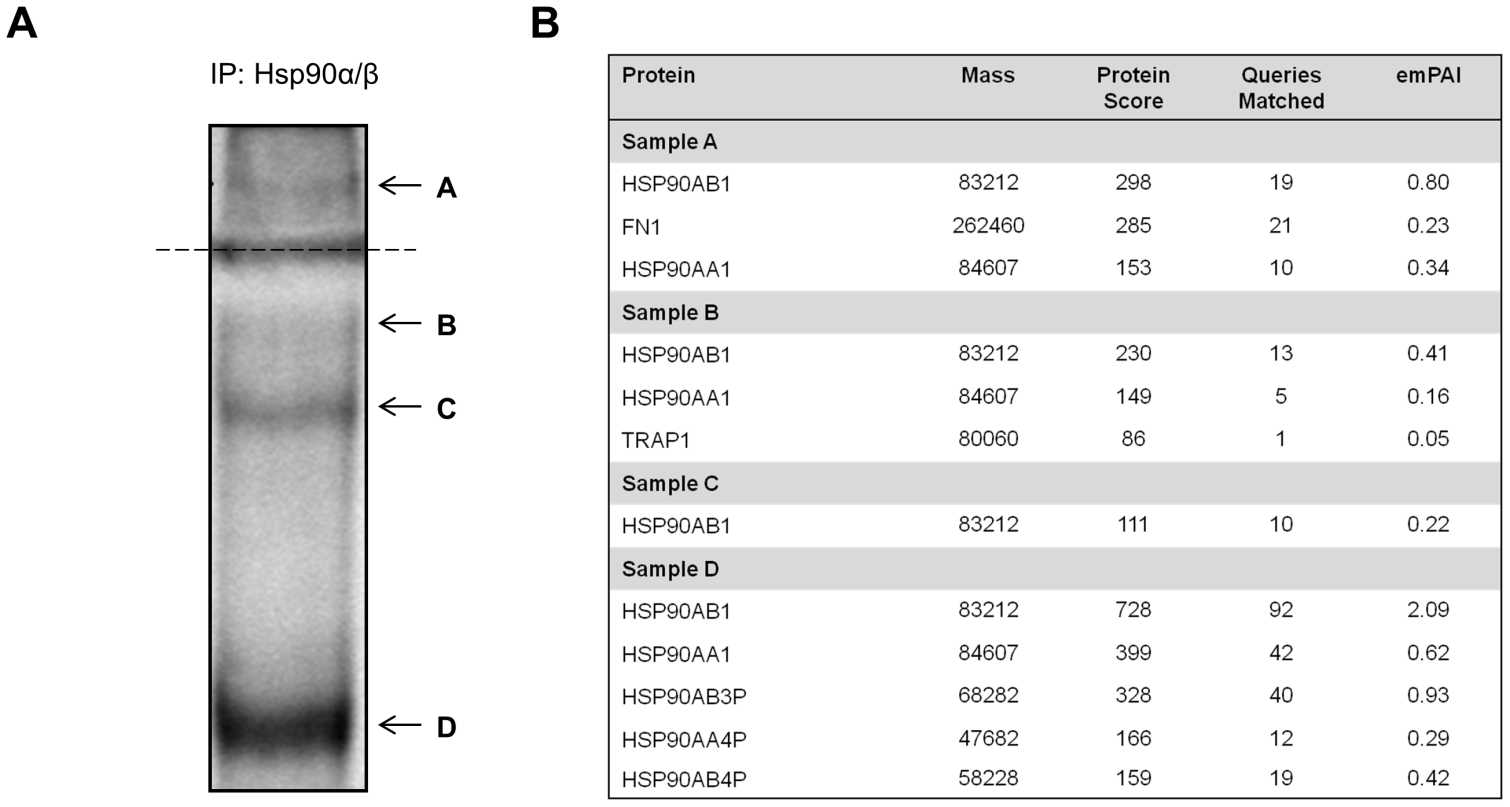
Identification of an extracellular Hsp90 complex containing the ECM protein FN. (**A**) Surface proteins of MDA-MB-231 cells were chemically crosslinked with the thiol-cleavable DTSSP reagent and cell lysates immunoprecipitated with Hsp90α/β primary antibody. Crosslinked samples were resolved by non-reducing SDS-PAGE followed by colloidal Coomassie staining. Four bands (labeled A–D) were excised from the gel and analysed by tandem mass spectrometry (MS/MS). The dotted line represents the stacking and resolving gel interface. (**B**) Data summary from the MS/MS analysis indicated the presence of Hsp90α/β (Band/sample A–D) and human FN1 (Band/sample A). The mass refers to the molecular weight of the respective proteins (in Daltons). A higher protein score indicates a more confident match. The queries matched refer to the number of sequences with a direct match to the query protein. The exponentially modified protein abundance index (emPAI) refers to the relative abundance of the query protein in each sample.

We subsequently conducted mass spectrometry to identify proteins in the isolated crosslinked Hsp90 containing complexes (Figure 2). Following a large scale crosslinking and immunoprecipitation using anti-Hsp90 antibody, samples were resolved by non-reducing SDS-PAGE. Equivalent control IP reactions using BSA-conjugated beads were conducted and no non-specific bands were detected by SDS-PAGE analysis (*data not shown*). Three higher molecular weight bands (Figure 2A; Bands A–C) and one band at the subunit molecular weight of Hsp90 (Figure 2A; Band D) were detected by colloidal Coomassie staining in IP samples using anti-Hsp90 antibody. Bands B, C and D appeared sufficiently resolved within the resolving gel, while Band A remained in the stacking gel. The individual bands were excised from the gel and analyzed by tandem mass spectrometry (MS/MS) (Figure 2B). The presence of specific proteins was determined using Mascot, which compares the observed spectra of ion data to the human taxonomy of the Swissprot database in order to determine most likely matches [47]. A good match arises if a query shows a high protein score, above the threshold, with multiple query matches [47]. The protein score is representative of the calculated probability that the observed match between the experimental data and database sequence is a random event for each distinct sequence [47]. All matched proteins in Figure 2B showed high significant protein scores for Hsp90α or Hsp90β indicating that Hsp90α and Hsp90β were present in all co-immunoprecipitated complexes. Sample A (corresponding to Band A on the gel), in addition to Hsp90, also contained peptides that matched human FN1 (FN) with a significantly high protein score and high number of queries matched. Upon inspection of the peptide summary report of Sample A, 17 individual ion scores of the 21 matched queries for FN1 were deemed significant (*data not shown*). Within Sample A, Hsp90β showed a higher protein score, queries matched score and higher relative abundance or emPAI score in comparison to Hsp90α. These data suggested that Hsp90α and Hsp90β may be in a common complex with FN on the surface of MDA-MB-231 cells.

### Hsp90 and FN Interact Directly *in vitro*

**Figure 3:**
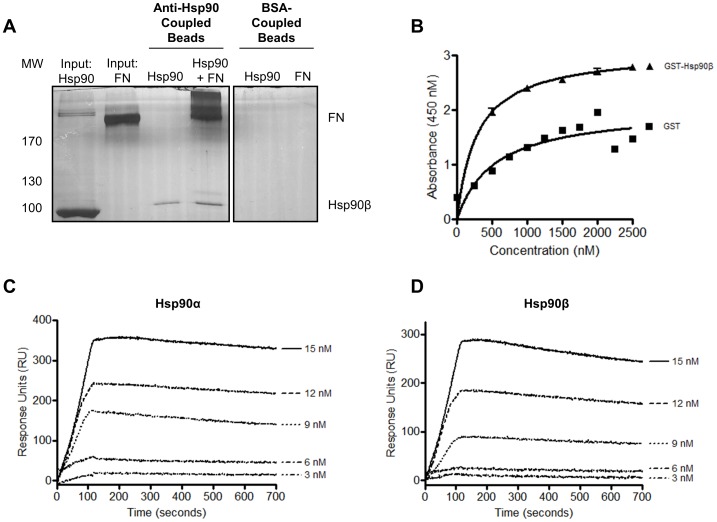
Hsp90β bound directly to FN *in vitro*. (**A**) Anti Hsp90β or BSA-coupled Dynabeads were incubated with Hsp90β and soluble FN and co-immunoprecipitated samples resolved by SDS-PAGE and analyzed by silver staining. Hsp90β and FN were loaded as input controls. (**B**) GST-Hsp90β and GST (negative control) bound adsorbed FN in a solid phase binding assay. Data analysis was performed in GraphPad Prism 4.03, Graphpad Software, Inc. Absorbance values were plotted versus concentration and a non-linear fit (one site binding; hyperbola) showed a R^2^ value equal to 0.7670 and 0.9481 for GST and GST-Hsp90β respectively. An F-test showed significant difference in the curve fits of GST and GST-Hsp90β with P<0.0001. Representative sensorgrams of observed binding of FN to immobilized (**C**) Hsp90α and (**D**) Hsp90β (10 µg.ml^−1^). Sensorgrams are representative of three experiments at varying FN concentrations.

The direct binding of Hsp90β to FN was investigated using three techniques, namely co-immunoprecipitation, solid phase binding assay and SPR spectroscopy (Figure 3). The ability of Hsp90β and FN to bind in solution was investigated by co-immunoprecipitation (Figure 3A). Recombinant Hsp90β and FN were included as input controls for the sizing of immunoprecipitates. Incubation of Hsp90β with anti-Hsp90α/β antibody-coupled Dynabeads® resulted in a single band at 90 kDa being observed, indicating successful immunoprecipitation. Upon incubation of Hsp90β with FN, a band at the molecular weight of FN co-immunoprecipitated with Hsp90β. Co-precipitation of FN with Hsp90β was verified both by silver staining (Figure 3A) and immunoblot analysis (*data not shown*). Incubation of BSA coupled Dynabeads® with Hsp90 or FN served as a negative control for non-specific binding.

GST-Hsp90β and GST (negative control) were expressed in *Escherichia coli* BB1994 cells and purified using an adapted GST purification protocol [48]. Protein purification was confirmed by immunoblotting (Figure S1A–C). The interaction of GST-Hsp90β to FN coated on a microtitre plate was determined by solid phase binding assay (Figure 3B). GST-Hsp90β or GST were incubated with FN (100 µg.ml^−1^) that had been adsorbed on the microtitre plate. Validation of adsorption of FN to the ELISA plate surface was carried out using a FN specific antibody (*data not shown*). There was an increase in absorbance with increasing concentrations of GST-Hsp90β (0–2500 nM), correlating to increased binding of GST-Hsp90β to adsorbed FN (Figure 3B). This suggested a concentration dependant interaction between GST-Hsp90β and FN with saturation observed. Non-specific binding of GST to FN was observed but this was below the level of GST-Hsp90β binding. A non-linear regression curve fit of the data resulted in r^2^ values of 0.7670 and 0.9481 for GST and GST-Hsp90β respectively. An F-test showed a statistically significant difference in the curve fits of GST and GST-Hsp90β (p<0.0001). A similar trend of interaction was observed with lower concentrations of adsorbed FN (10 ug.ml^−1^) (*data not shown*).

With an initial interaction observed, binding of Hsp90α and Hsp90β to FN was determined by SPR spectroscopy using the ProteOn™ XPR36 Protein Interaction Array System (Figure 3C and D). Representative sensorgrams demonstrated a dose dependent interaction between FN in solution and immobilized Hsp90α and Hsp90β (10 µg.ml^−1^). Saturated binding of FN to Hsp90 was observed at higher analyte concentrations (Figure S1D). R_eq_ values were plotted against concentration of FN and a binding saturation curve generated (Figure S1E). Hsp90β was also shown to bind immobilized FN in a reciprocal experiment (*data not shown*).

### Hsp90 Colocalised with FN in Cancer and Normal Cell Lines

**Figure 4:**
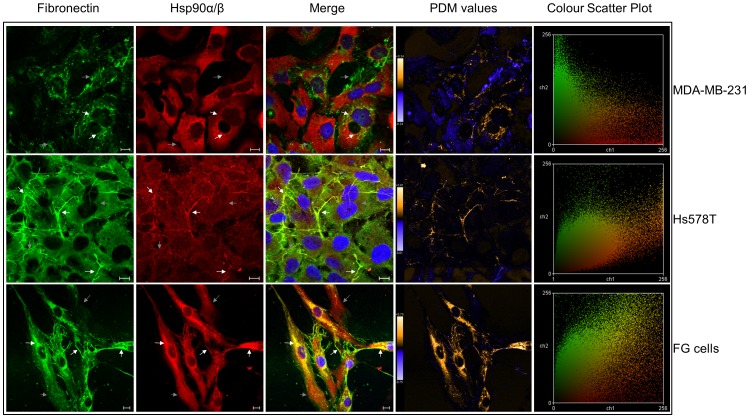
Hsp90α/β and FN localisation in MDA-MB-231, Hs578T and FG cells. Fixed MDA-MB-231, Hs578T and FG cells stained using mouse anti-human FN and goat anti-human Hsp90α/β followed by donkey anti-mouse DyLight® 488 and donkey anti-goat DyLight® 650 fluorescent secondary antibodies, respectively. Nuclei were stained with Hoechst 33342 (1 µg.ml^−1^). Images were captured using a Zeiss LSM 510 Meta laser scanning confocal microscope and analyzed using Zen, blue edition, Zeiss, Germany. White arrows show regions of interest for co-localisation, gray arrows show regions where co-localisation is not prevalent. PDM values and a Colour Scatter Plot were generated using Image J. Positive and negative PDM values are indicated in orange and blue respectively. Scale bars are equivalent to 10 µm. The data shown are representative of triplicate experiments.

Having confirmed the direct interaction of Hsp90β with FN *in vitro*, we investigated the localisation of these proteins in normal and cancer cell lines using confocal microscopy (Figure 4). Hsp90α/β and FN expression was detected in the breast cancer lines MDA-MB-231 and Hs578T, as well as the primary fibroblast FG cell line. Merged images showed overlapping green and red intensities. The PDM values show the product of the differences from the mean for each pixel, with highlighted PDM values showing regions of possible co-localisation [49]. The Colour Scatter Plot generated shows a plot of green intensities (FN) vs. red intensities (Hsp90α/β) with yellow indicative of overlapping green and red pixels. FN is secreted by cells as a soluble dimer that is then assembled into an insoluble network of fibres in the extracellular space [50]. Degradation of this extracellular FN matrix occurs intracellularly after endocytosis [41]. FN was thus expected to be present both in an intracellular form and extracellularly as a fibrillar network. The MDA-MB-231 cell line showed lower levels of extracellular fibrillar FN in comparison to the Hs578T and FG cell lines. A dispersed intracellular FN localisation pattern was observed in MDA-MB-231 cells. The lower expression level of FN in MDA-MB-231 cells in comparison to Hs578T cells was confirmed by immunoblotting of cell lysates (*data not shown*). Hsp90α and Hsp90β have been shown to reside both in the cytosol [14], and the extracellular environment [17], [18], [19]. This was confirmed with dispersed staining patterns seen for Hsp90 in all three cells lines. Hsp90, in MDA-MB-231 cells, showed a diffuse localisation pattern distinct from FN, although selected regions of co-localisation were identified (Figure 4, white arrows). Hs578T cells showed intracellular staining for FN and Hsp90α/β, as well as predominant extracellular FN fibrils. Hsp90α/β, in Hs578T cells, appeared to localise distinctly with selected extracellular FN fibrils as seen in the PDM values output. More defined co-localisation was observed with larger fibrils suggesting an interaction between extracellular Hsp90 and the FN matrix. Although isolation and identification of an extracellular Hsp90 complex occurred from MDA-MB-231 cells, lower levels of extracellular FN matrix in this cell line were observed, making it difficult to conclude that Hsp90 co-localised solely with existing extracellular FN fibrils in this cell line. The localisation patterns of FN and Hsp90α/β were similar in FG cells. Hsp90α/β showed definitive perinuclear and possible Golgi co-localisation with intracellular FN. Co-localisation of Hsp90α/β with extracellular FN fibrils was also observed, however to a lesser extent than seen in the Hs578T cells.

### Exogenous Hsp90β Increased the Formation of Extracellular FN Matrix

**Figure 5:**
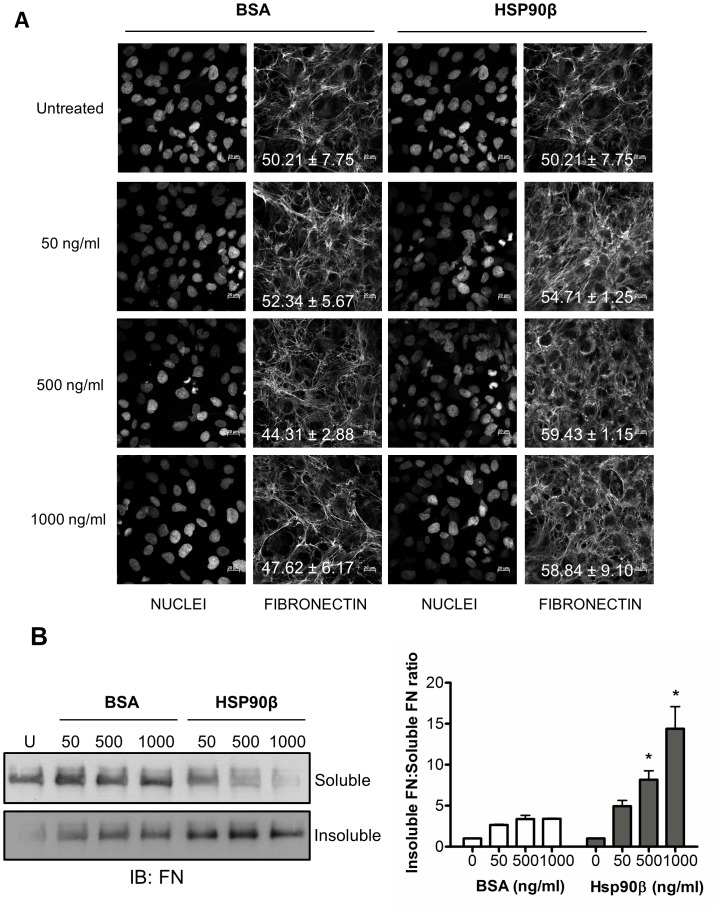
Exogenous Hsp90β increases the proportion of insoluble FN in Hs578T cells. (**A**) Adherent Hs578T cells were treated with exogenous endotoxin-free BSA (negative control) or Hsp90β at increasing concentrations (50, 500 and 1000 ng.ml^−1^) and the effect on the FN matrix examined by confocal microscopy by staining using mouse anti human FN followed by donkey anti mouse DyLight® 488 secondary antibody. The proportion of FN staining in the confocal images was quantified by calculating the mean gray value across all images using ImageJ. The average mean gray values and standard deviation derived from triplicate treatments are indicated on the images in white text. (**B**) Changes in DOC-soluble and DOC-insoluble FN levels were detected by immunoblotting using mouse anti human FN antibody. Equal amounts of total protein were loaded for each sample as determined by A_280nm_. The densitometry for each sample was determined using ImageJ and ratio of insoluble FN to soluble FN compared for respective treatments. Values are averages obtained from three independent experiments. Statistical significance was determined by one-way ANOVA (*p<0.01) comparing BSA and Hsp90β treated samples at the equivalent concentrations.

Following an interaction observed *in vitro* and colocalization of Hsp90 and FN in both normal and cancer cell lines, we next investigated the effect of exogenous Hsp90β on FN matrix formation and morphology in Hs578T cells using confocal microscopy and the DOC assay (Figure 5A and B respectively). A change in the morphology of the extracellular FN matrix was detected by confocal microscopy in cells treated with Hsp90β compared to untreated cells or cells treated with BSA (Figure 5A). An increase in the average mean gray value upon treatment with exogenous Hsp90β was observed. Cells treated with Hsp90β consisted of insoluble fibers that appeared finer and more dispersed between cells. Nuclei stained with Hoechst 33342 ensured the observed phenotype was not a result of a different cell numbers.

The DOC assay [40], was performed in order to fractionate DOC-soluble (cell-associated) and DOC-insoluble (matrix-associated) FN fractions. As a means to validate this assay in our cell line, Hs578T cells treated with the MEK inhibitor U0126 were lysed and immunoblotting showed FN but not GAPDH (intracellular protein) was included in the DOC-insoluble fraction (Figure S2). The addition of exogenous BSA did not result in a dramatic increase in the proportion of DOC-insoluble FN isolated from the Hs578T cell line (Figure 5B). The addition of exogenous soluble Hsp90β led to a dose-dependent, statistically significant, increase in the proportion of insoluble FN, accompanied by an apparent decrease in the levels of soluble FN (Figure 5B).

### Knockdown of Hsp90α and Hsp90β Reduced FN Levels

**Figure 6:**
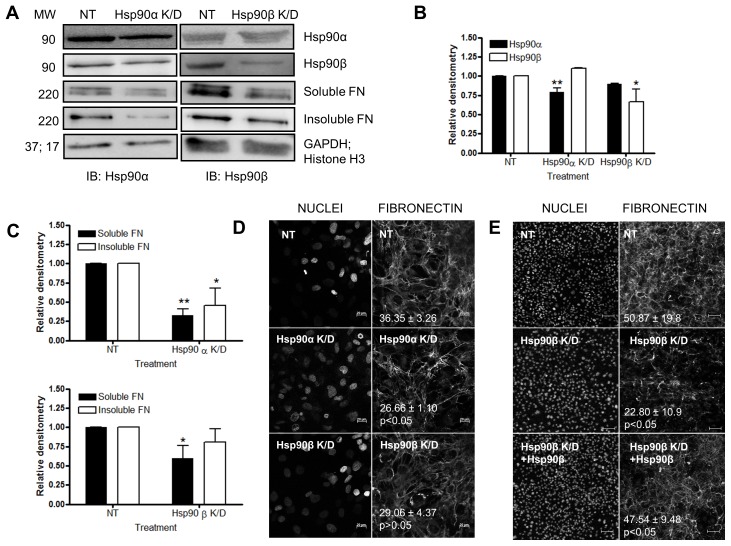
Knockdown of Hsp90α or Hsp90β with siRNA decreased the proportion of extracellular FN matrix. (**A**) Hs578T cells were transfected with a pool of siRNA against either Hsp90α (Hsp90α K/D) or Hsp90β (Hsp90β K/D). Scrambled non-targeting (NT) siRNA served as a negative control. After 48 hours, levels of Hsp90α and Hsp90β were analysed by immunoblotting with mouse anti human Hsp90α or Hsp90β primary antibodies respectively. Transfected cells were harvested and DOC-soluble and DOC-insoluble fractions separated. Levels of DOC-soluble and DOC-insoluble FN were analyzed by immunoblotting with mouse anti human FN primary antibody. Levels of GAPDH or Histone H3 were used to ensure equal sample loading (**B**) Relative densitometry of expression levels of Hsp90α and Hsp90β of NT and Hsp90 knockdown (K/D) transfected Hs578T cells, determined by Image J 1.43m. (**C**) Relative densitometry of levels of soluble and insoluble FN of NT and Hsp90 K/D transfected Hs578T cells, determined by Image J. (**D**) Transfected Hs578T cells were fixed and stained with mouse anti human FN followed by donkey anti mouse DyLight® 488 secondary antibody. Nuclei were stained with Hoechst 33342 (1 µg.ml^−1^). Images were captured using a Zeiss LSM 510 Meta laser scanning confocal microscope and analyzed using AxiovisionLE 4.7.1, Zeiss, Germany. Confocal microscopy images for each treatment were captured in triplicate and values in white represent the mean grey values (± standard deviation) for each treatment. Scale bars are equivalent to 20 µm. The data shown are from triplicate experiments. (E) Exogenous Hsp90β partially recovered the FN phenotype in Hsp90β knockdown cells. Adherent Hs578T cells were treated with siRNA against Hsp90β and then remained untreated or were treated with Hsp90β (100 ng.ml^−1^). Cells were fixed and stained for FN as described previously. Scale bars are equivalent to 100 µm. The average mean gray values and standard deviation from 5 different fields of view are indicated on the images in white text. Statistical analysis was performed by comparing siRNA treated cells to cells treated with siRNA and exogenous Hsp90β, using one way ANOVA with Bonferroni post-test. Images shown are representative of duplicate experiments.

We next investigated the effect of transient silencing of Hsp90α or Hsp90β in Hs578T cells on the levels of DOC-soluble and DOC-insoluble FN fractions (Figure 6). Approximately 30% knockdown was achieved for Hsp90α with little to no effect on the levels of Hsp90β. Similarly approximately 40% knockdown was achieved for Hsp90β with little to no effect on the levels of Hsp90α (Figure 6A and B). These reductions in Hsp90α and Hsp90β did not result in a substantial decrease in cell viability (*data not shown*). Cells transfected with a pool of scrambled, non-targeting siRNA oligonucleotides acted as a control. The levels of DOC-soluble and DOC-insoluble FN were analyzed by immunoblotting in cells treated with Hsp90 specific siRNA. Knockdown of either Hsp90α or Hsp90β resulted in significant reductions in the levels of soluble and insoluble FN respectively (Figure 6A and C). A decrease in the FN extracellular matrix of Hs578T cells was confirmed by confocal microscopy where a decrease in the mean gray value from 36.35±3.26, for the non-targeting control, to 26.66±1.10, for Hsp90α knockdown and 29.06±4.37 for Hsp90β knockdown was observed (Figure 6D). The FN phenotype in knockdown cells was rescued by the addition of exogenous extracellular Hsp90β (Figure 6E). A significant (p<0.05) increase in the mean gray value for FN staining was observed in Hsp90β siRNA treated cells upon addition of exogenous Hsp90β (47.54±9.48), compared to cells treated with siRNA against Hsp90β alone (22.80±10.9). In all cases, cells transfected with a pool of scrambled, non-targeting siRNA oligonucleotides were used as the control and did not show any substantial changes in the levels of Hsp90 or FN compared to untreated cells.

### Inhibition of Hsp90 with Novobiocin Reduced Extracellular FN Matrix

**Figure 7:**
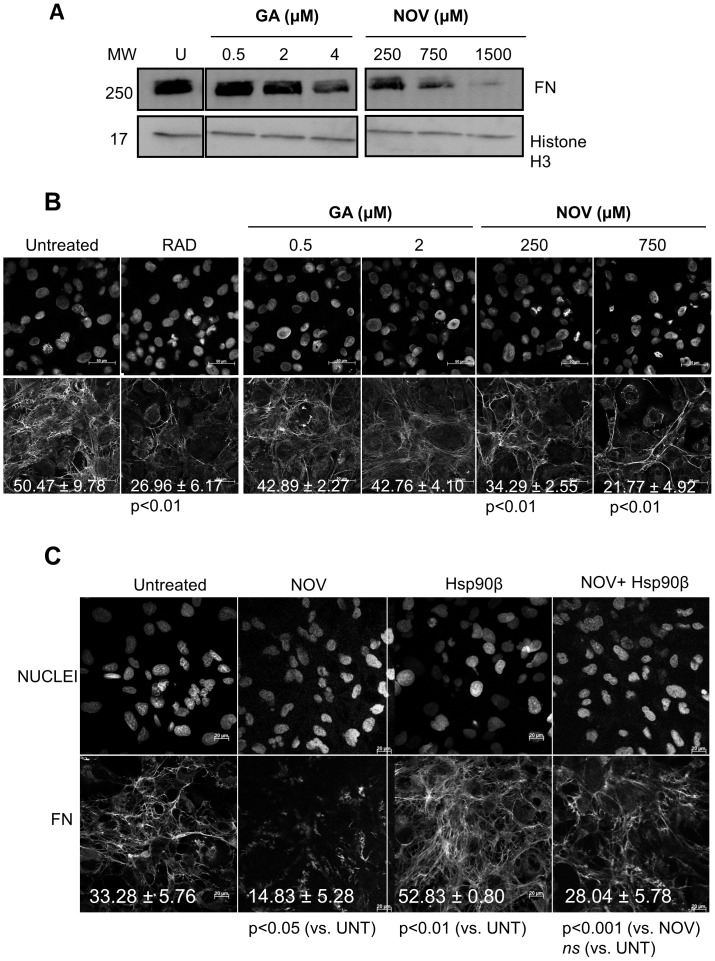
Inhibition of Hsp90 with novobiocin reduced the extracellular FN matrix. (**A**) Adherent Hs578T cells were treated with increasing doses of geldanamycin (GA; 0.5, 2 and 4 µM) or novobiocin (NOV; 250, 750 and 1500 µM) for 16 hours. Cells were lysed and equal amounts of total cell protein probed for FN by immunoblotting using mouse anti human FN. A probe for Histone H3 served as a loading control. Data are representative of three independent experiments with similar results. (**B**) Adherent Hs578T cells were treated with RAD peptide (5 µg.ml^−1^) or increasing doses of geldanamycin (GA; 0.5 and 2 µM) or novobiocin (NOV; 250 and 750 µM) for 16 hours. Cells were fixed and stained using mouse anti human FN followed by anti mouse Alexa-Fluor-488 secondary antibody. Scale bars are equivalent to 50 µm. The proportion of FN staining in the confocal images was quantified by calculating the mean gray value across all images using ImageJ. The average mean gray values and standard deviation derived from replicate treatments are indicated on the images in white text. Statistical analysis was performed by comparing all samples with the untreated control using one way ANOVA with Bonferroni post-test. Values shown are averages of three independent experiments. (**C**) Exogenous Hsp90β partially recovered the novobiocin treated extracellular FN matrix. Adherent Hs578T cells were treated with novobiocin (NOV; 250 µM), Hsp90β (100 ng.ml^−1^) or a combination of both NOV and Hsp90β for 16 hours. Cells were fixed and stained using mouse anti-human FN followed by anti-mouse Alexa-Fluor-488 secondary antibody. Scale bars are equivalent to 20 µm. The proportion of FN staining in the confocal images was quantified by calculating the mean gray value across all images using ImageJ. The average mean gray values and standard deviation derived from replicate treatments are indicated on the images in white text. Statistical analysis was performed by comparing NOV and Hsp90β treated samples with the untreated control, and NOV in combination with Hsp90β treatment to NOV treatment alone, using one way ANOVA with Bonferroni post-test. Values shown are averages of three independent experiments.

We next tested the effect of published inhibitors of Hsp90, geldanamycin (GA) and novobiocin (NOV), on the extracellular FN matrix (Figure 7A and B). Experiments were conducted at inhibitor concentrations at which the Hs578T cell line displayed greater than 90% cell viability throughout the experiment (*data not shown*). A dose-dependent decrease in the levels of total FN was observed upon treatment of cells with Hsp90 inhibitors (Figure 7A). FN levels were only affected at high concentrations of GA but NOV treatment led to a significant decrease in the levels of total FN compared to the untreated cells (Figure 7A). Confocal microscopy also showed a statistically significant decrease in the extracellular FN matrix in cells treated with NOV (p<0.01) compared with untreated cells (Figure 7B). Treatment with GA led to a change in matrix phenotype demonstrating a more ordered fibrillar network relative to untreated cells, although a prominent extracellular matrix was still present (Figure 7B). The FN phenotype in NOV treated cells was similar to the FN matrix morphology in cells treated with an RAD peptide. The RAD peptide is an RGD analogue that inhibits FN matrix formation through competition with FN for integrin binding [51], and served as a positive control for a loss in extracellular FN matrix assembly. Cells treated with the topoisomerase II inhibitor, etoposide [52] maintained a substantial FN matrix, showing that the effect of novobiocin inhibition was not as a result of inhibition of topoisomerase II [53] (Figure S3).

We tested whether the addition of exogenous soluble Hsp90β was capable of rescuing the loss of FN observed in NOV treated cells (Figure 7C). To ensure the phenotypes were not a result of reduced cell number, nuclei stained with Hoechst 33342 were counted relative to untreated cells. Confocal microscopy confirmed the statistically significant increase in the insoluble FN matrix observed in Hs578T cells treated with Hsp90 (100 ng/ml) when compared to the untreated cells. Cells treated with NOV alone (250 µM) showed a loss in the extracellular FN matrix. Cells treated with NOV (250 µM) and Hsp90β (100 ng/ml) showed a statistically significant increase in the extracellular FN matrix compared to cells treated with 250 µM NOV alone. The FN matrix in NOV and Hsp90 treated cells was not significantly different from untreated cells. This suggested that addition of extracellular Hsp90 was sufficient to partially rescue the extracellular FN phenotype in NOV treated cells.

### Inhibition of Hsp90 with Novobiocin Results in FN Internalization

**Figure 8:**
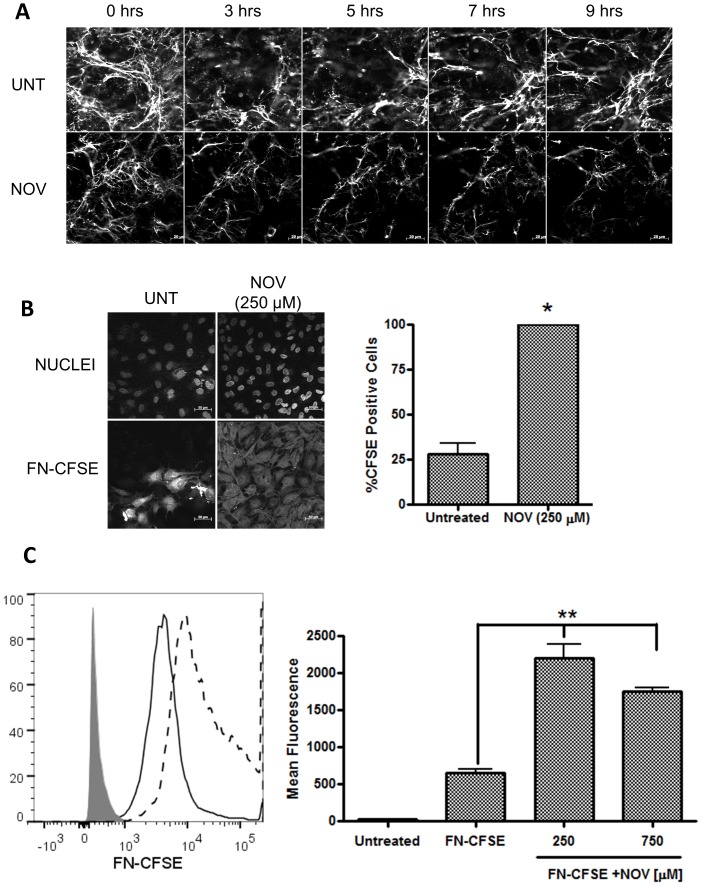
Novobiocin treatment resulted in FN internalization. (**A**) Hs578T cells were grown in phenol-red free media in sterile glass-bottomed microscopy culture dishes and allowed to undergo fibrillogenesis in media supplemented with fluorescently labelled FN (50 nM). Following the confirmation of a fluorescent extracellular FN matrix, cells either remained untreated or were treated with novobiocin (NOV; 1 mM) and live cell images captured every 15 minutes over a period of 9 hours using confocal microscopy. Images show single frames from the live cell experiment captured at specific time points. Scale bars are equivalent to 20 µm. (**B**) Adherent Hs578T cells were treated with either FN-CFSE (500 nM) alone or in combination with novobiocin (NOV; 250 µM) for 16 hours. Cells were fixed and images were captured using confocal microscopy. Three images were captured for each treatment in areas where similar cell numbers were observed. Scale bars are equivalent to 50 µm. The proportion of cells stained positive for CFSE, calculated by dividing the total number of cells positive for fluorescence by the total number of cells (nuclei) in each frame are shown alongside. Data are representative of three independent experiments with similar results. Statistical analysis was performed by comparing NOV (250 µM) treated samples to the untreated control using one way ANOVA with Bonferroni post-test (*p<0.01) (**C**) Hs578T cells were treated with either FN-CFSE (500 nM) alone or in combination with novobiocin (250 and 750 µM). Relative fluorescence of untreated cells (grey shading), FN-CFSE treated cells (solid line) and novobiocin (250 µM) treated cells (dashed line) was determined by flow cytometry using the FITC channel on a FACSAria II instrument. The average mean fluorescence from triplicate treatments was quantified using FlowJo (TreeStar Inc) and normalised for background fluorescence. Statistical significance relative to cells treated with FN-CFSE alone was determined using a one-way ANOVA with Bonferroni post-test (**p<0.001). Data are averages of three independent experiments.

Given that inhibition of Hsp90 with NOV resulted in a reduction of extracellular FN matrix in Hs578T cells, we next investigated whether this reduction was as a result of internalization of only extracellular FN by monitoring the effect of NOV on exogenous, fluorescently labelled FN (Figure 8). Confocal microscopy revealed that untreated Hs578T cells successfully incorporated fluorescently labelled FN into an extracellular matrix (Figure S4A). The effect of NOV on the extracellular fluorescent FN matrix of Hs578T cells was subsequently analyzed using live cell imaging over a period of 9 hours (Figure 8A) or a pulse chase assay (Figure S4B). Cells were treated with NOV after the presence of a fluorescent FN matrix was confirmed. Untreated cells showed changes in matrix morphology due to FN matrix turnover and assembly, but no considerable change in matrix density was seen (Figure 8A – UNT). Upon treatment with NOV, a time-dependent decrease in the fluorescent FN matrix was observed (Figure 8A – NOV). To test whether the decrease in the fluorescent FN matrix was due to internalization of FN, Hs578T cells were treated with FN conjugated to the dye CFSE (FN-CFSE) alone or in combination with NOV and the fluorescent staining pattern observed by confocal microscopy (Figure 8B). CFSE is a non-fluorescent dye when extracellular and becomes fluorescent when cleaved by intracellular esterases. Therefore only FN-CFSE that has been internalized should become fluorescent [54]. A greater proportion of cells were positive for CFSE fluorescence when treated with FN-CFSE in combination with NOV as opposed to cells treated with FN-CFSE alone (Figure 8B). The intensity of CFSE fluorescence (indicating the proportion of FN internalized by the cells) was determined by flow cytometry. Treatment with NOV led to a statistically significant increase in FN-CFSE fluorescence in comparison to untreated cells or cells treated with FN-CFSE alone (Figure 8C). In all cases we did not observe any fluorescent extracellular matrix, only intracellular FN-CFSE fluorescence. The internalisation was specific to FN as BSA labelled with CFSE did not result in detectable intracellular fluorescence (*data not shown*).

### FN was Targeted to LAMP1 Positive Vesicles upon Novobiocin Treatment

**Figure 9:**
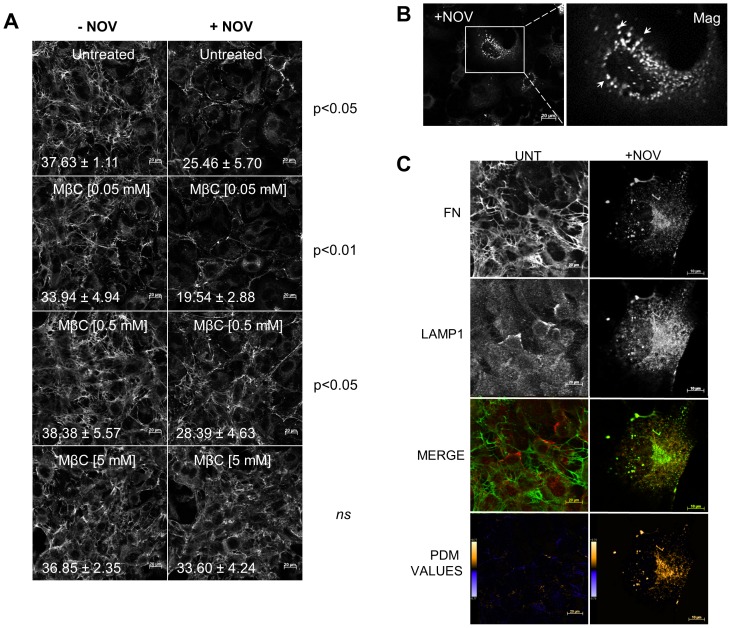
Inhibition of receptor mediated endocytosis reversed the effect of novobiocin on the extracellular FN matrix. (**A**) Adherent Hs578T cells were treated with increasing concentrations of methyl-β-cyclodextrin (MβCD) and incubated for 1 hour prior to treatment with novobiocin (1 mM) for 7 hours at 37°C. Cells treated with MβCD alone served as a control. FN was detected by staining with mouse anti human FN followed by donkey anti mouse Alexa-Fluor-488 fluorescent secondary antibody. Images were captured using confocal microscopy. Scale bars are equivalent to 20 µm. The proportion of FN staining was quantified by calculating the mean gray value across all images using ImageJ. The average mean gray values and standard deviation derived from replicate treatments are indicated on the images in white text. Data are representative of three independent experiments with similar results. Statistical analysis was performed by comparing MβCD treated samples with the MβCD and novobiocin treated samples using one way ANOVA with Bonferroni post-test. (**B**) Representative magnified image of punctate intracellular FN staining pattern (white arrows) observed in novobiocin treated Hs578T cells. Scale bar is equivalent to 20 µm. (**C**) Adherent Hs578T cells were untreated or treated with novobiocin (250 µM) for 16 hours at 37°C. Cells were fixed and FN and LAMP1 detected by staining with rabbit anti human FN and mouse anti human LAMP1 primary antibody followed by donkey anti rabbit Alexa-Fluor-488 (green) and donkey anti-mouse Alexa-Fluor 633 (red) fluorescent secondary antibodies respectively. Data are representative of three independent experiments with similar results. The graph of PDM values was generated using Image J in which positive and negative PDM values are indicated in orange and blue respectively.

As FN internalisation has been shown to occur via endocytosis, we investigated whether the internalisation of FN in response to NOV treatment could be reversed by inhibitors of vesicle trafficking. A panel of different endocytosis inhibitors (colchicine, genistein, cytochalasin B and methyl-β-cyclodextrin) were tested for their ability to block the effect of NOV treatment on FN (*data not shown*). Treatment of Hs578T cells with methyl-β-cyclodextrin (MβCD), an inhibitor of receptor mediated endocytosis, led to a dose-dependent and statistically significant reversal of the FN matrix phenotype in the presence of NOV but not when used alone (Figure 9A). Closer inspection of the FN matrix in NOV treated cells showed the presence of vesicular like structures within the cytoplasm of treated cells (Figure 9B). Hs578T cells treated with NOV were stained for FN and the lysosomal marker LAMP1. Results indicated poor co-localization between FN and LAMP1 in untreated cells (Figure 9C). Treatment with NOV, however, resulted in an increase in the co-localisation between FN and LAMP1 staining (Figure 9C). The degree of colocalisation was quantified using Pearson’s correlation coefficient (R) calculated with ImageJ [18]. There was an increase in the Pearson’s coefficient in cells treated with NOV (0.881±0.039 for LAMP1 and FN) relative to untreated cells (0.203±0.089 for LAMP1 and FN).

## Discussion

This is the first study to demonstrate that Hsp90 and FN interact directly and that inhibition of Hsp90 resulted in changes in the extracellular FN matrix. FN is secreted by cells as a soluble dimer that is then assembled into an insoluble network of fibers [50], [55]. The binding of FN to cell surface integrins initiates conformational changes in the FN molecule that exposes self-association sites and the formation of an insoluble FN matrix in the extracellular space [30], [50], [55]. Treatment of the Hs578T cell line with exogenous soluble Hsp90β led to an increase in insoluble FN relative to soluble FN, suggesting a role for extracellular Hsp90 in fibril formation. Hsp90 has been previously shown to promote fibrillogenesis by α-synclein by stabilizing intermediate conformations of the protein during fibril assembly [56]. The subsequent remodelling of the ECM is a highly regulated process involving the interplay between extracellular matrix synthesis, deposition and degradation [40]. Any stimulus that inhibits fibrillogenesis will promote the turnover of FN, enhancing FN endocytosis and intracellular degradation [41]. Inhibition of Hsp90 with novobiocin led to a destabilisation of the matrix resulting in FN internalisation and degradation in lysosomes. Matrix instability has previously been shown to result in FN degradation in the lysosome via a caveolin-1 dependent endocytic pathway [41]. The effects of novobiocin were not mediated by inhibition of topoisomerase II, as etoposide failed to induce a similar response. Addition of exogenous Hsp90β or MβCD to cells was able to reverse at least some of the effects on the FN matrix of Hsp90 inhibition. Taken together, these data may be interpreted to mean that Hsp90 is involved in processes that lead to the formation of insoluble FN from soluble FN, as well as maintaining the stability of the extracellular matrix. As a molecular chaperone, Hsp90 is responsible for maintaining the integrity of numerous client proteins through regulation of protein conformation and stability [14], [57], [58], [59]. A client protein of Hsp90 can be defined by two criteria, firstly that it should be capable of direct association with the chaperone, and secondly it should be dependent on the interaction with Hsp90 for stability or conformational regulation [60], such that Hsp90 inhibition leads to its destabilization and degradation [27]. Based on the direct interaction between the two molecules and the degradation of FN in response to pharmacological inhibition of Hsp90, we propose that FN is a putative client protein of Hsp90.

The differential effects of geldanamycin and novobiocin on the FN matrix may be explained by the mechanism by which these compounds inhibit Hsp90. Geldanamycin is known to bind the N-terminal ATPase domain of intracellular Hsp90, inhibit ATP binding and ATP-driven N-terminal dimerisation of Hsp90, thereby blocking the formation of the active conformation [61]. Novobiocin, in contrast, binds the C-terminal pocket of Hsp90, inhibits Hsp90 dimerization and competes with co-chaperones for binding to Hsp90 [62], [63]. The concentration of ATP in the extracellular environment is likely to be below the threshold for binding to Hsp90 and therefore it is possible that the extracellular functions of Hsp90 may independent of ATP-mediated N-terminal dimerisation, substantiating why geldanamycin did not have a dramatic effect on the FN matrix.

The mechanism by which Hsp90 affects the FN matrix is presently uncharacterised. Chen and colleagues recently showed that extracellular Hsp90α could induce FN expression by activating cellular signalling pathways in transcription factor 12 (TCF-12) overexpressing colorectal cancer cell lines [29]. Therefore Hsp90 inhibition may reduce FN expression, while the effects of exogenous Hsp90 may be due to an increase in FN expression in breast cancer. Despite the evidence for direct interaction between Hsp90 and FN *in vitro*, it is also possible that some of the effects of Hsp90 knockdown or inhibition on the FN matrix are indirect. FN matrix assembly is a complex process, dependent upon numerous other proteins, some of which are Hsp90 client proteins [40], [64]. Our data are still consistent with a role for both intracellular and extracellular Hsp90 in FN matrix dynamics. It is not unknown for molecular chaperones to exhibit concomitant intracellular and extracellular regulation of certain proteins. Hsp47, an intracellular chaperone, has been found in association with type IV collagen molecules on the surface of murine parietal endoderm cells [65]. With an established role in collagen biosynthesis along the secretory pathway, Hsp47 was shown to be exported as a complex where it was proposed to act as a receptor for collagen fibrillogenesis [65], [66], [67]. Furthermore, Hsp47 knockdown led to detrimental effects on both pro-collagen maturation and the insoluble collagen matrix suggesting a dependency on both intra- and extracellular Hsp47 [68]. Knockdown of the endoplasmic reticulum Hsp90 isoform, GRP94, in bone marrow hematopoietic stem cells (HSCs) led to a loss of expression of the α4-integrin and reduced binding to FN [69]. Likewise, inhibition of Hsp90 in the PC3 prostate cancer line led to reduced adhesion between FN and the β1-integrin [70]. We hypothesise that soluble FN may depend on intracellular Hsp90 for stability or transport prior to cellular export. Upon secretion, the activation and unfolding of soluble FN into an insoluble matrix may require extracellular and intracellular Hsp90 activity.

Irrespective of the mechanism, the fact that Hsp90 may influence FN function is an important finding, given that the dynamics of FN matrix assembly play a major role in cell migration and invasion. Increased turnover of the extracellular FN matrix in particular, has been correlated with enhanced metastatic capacity of tumour cells [71], [72], [73]. Hsp90 inhibition is considered a strategy for treatment of cancer and many Hsp90 inhibitors are in clinical trials. However, if inhibition of Hsp90 does lead to FN turnover, this may have consequences for cancer metastasis. Further clarification of the role of Hsp90 in FN matrix dynamics and description of the precise mechanism by which this occurs is ongoing.

## Supporting Information

Figure S1 **Hsp90β bound directly to FN in a dose dependant manner.** Immunoblot of GST and GST-Hsp90β purifications used in the solid phase binding assay. Overproduction of GST-Hsp90β and GST occurred using the *Escherichia coli* BB1994 (MC4100 *dnaK52 sidB1::Tc pDMI, 1::CmR KanR)* strain as a host. Purification of GST-Hsp90β and GST occurred according to an adapted protocol of the batch purification of GST-tagged proteins in the Protino® Glutathione Agarose (GSH) 4B (745500, Macherey-Nagel, Germany) user manual. Immunoblots were probed using (**A**)+(**B**) anti GST and (**C**) anti Hsp90β antibodies. Letters above each immunoblot represent the collected elution fractions. Arrows show the purified GST, GST-Hsp90β and GST-tagged degradation/truncation products. (**D**) Representative sensorgrams of observed binding of FN (20, 40, 80, 160 and 320 nM) to immobilized Hsp90β (10 µg.ml^−1^). Hsp90β was immobilized onto the surface of a ProteOn™ GLM Sensor chip (#176-5012, Bio-Rad, US) and protein interaction was determined at 37°C. Sensorgrams are representative of quadruple experiments at varying FN concentrations (**E**) Saturation binding curve of FN-Hsp90β SPR binding data. R_eq_ values were plotted against concentration and fit with a non-linear curve (one site binding). An R^2^ value equal to 0.9800 was reported.
(TIF)

Figure S2 **Validation of the deoxycholate (DOC) assay to quantitate FN matrix assembly in Hs578T breast cancer cells.** Confluent Hs578T cells were incubated with increasing concentrations of the MEK1/2 inhibitor (U1026) for 16 hours at 37°C. Cells were lysed and DOC-soluble and DOC-insoluble FN fractions isolated using the described DOC assay and relative levels determined by immunoblotting. Analysis of GAPDH levels demonstrated no contamination between the soluble and insoluble fractions.
(TIF)

Figure S3 **Etoposide has no effect on the extracellular FN matrix.** Adherent Hs578T cells were treated with etoposide (0.5 µM or 1 µM) and the effect on the FN matrix examined by confocal microscopy. The IC_50_ value (0.96 µM) for etoposide has previously been determined in the Hs578T cell line [1]. Duplicate images of etoposide (0.5 µM) treatment are shown. Scale bars are equivalent to 50 µm.
(TIF)

Figure S4 **Analysis of fibronectin dynamics using exogenous fluorescent FN.** (**A**) Cell imaging of exogenously added fluorescently labeled FN (FN-550). Hs578T cells were grown in phenol-red free media in sterile glass-bottomed microscopy culture dishes and allowed to undergo fibrillogenesis in media supplemented with FN-550 (50 nM). Fixed cells were stained using mouse anti human FN followed by donkey anti mouse DyLight® 488 fluorescent secondary antibodies. Images were captured using the Zeiss LSM 510 Meta confocal microscope. Scale bars are equivalent to 20 µm. White arrows show regions of incorporation of the exogenous FN-550 into the extracellular FN matrix. (**B**) Confluent Hs578T cells were allowed to undergo fibrillogenesis in media supplemented with FN-550 (50 nM). Following the confirmation of a fluorescent extracellular FN matrix, cells either remained untreated or were treated with novobiocin (NOV; 1 mM) for a period of 2, 4 or 8 hours. Cells were fixed and images captured using confocal microscopy. Three images were captured for each treatment in areas where similar cell numbers were observed. Scale bars are equivalent to 20 µm. Data are representative of three independent experiments with similar results.
(TIF)
