# Supplementary figures and images for: Hsp90 Binds Directly to Fibronectin (FN) and Inhibition Reduces the Extracellular Fibronectin Matrix in Breast Cancer Cells
Source: PLoS One. 2014 Jan 22;9(1):e86842. doi: 10.1371/journal.pone.0086842 (PMC3899338; doi:10.1371/journal.pone.0086842)

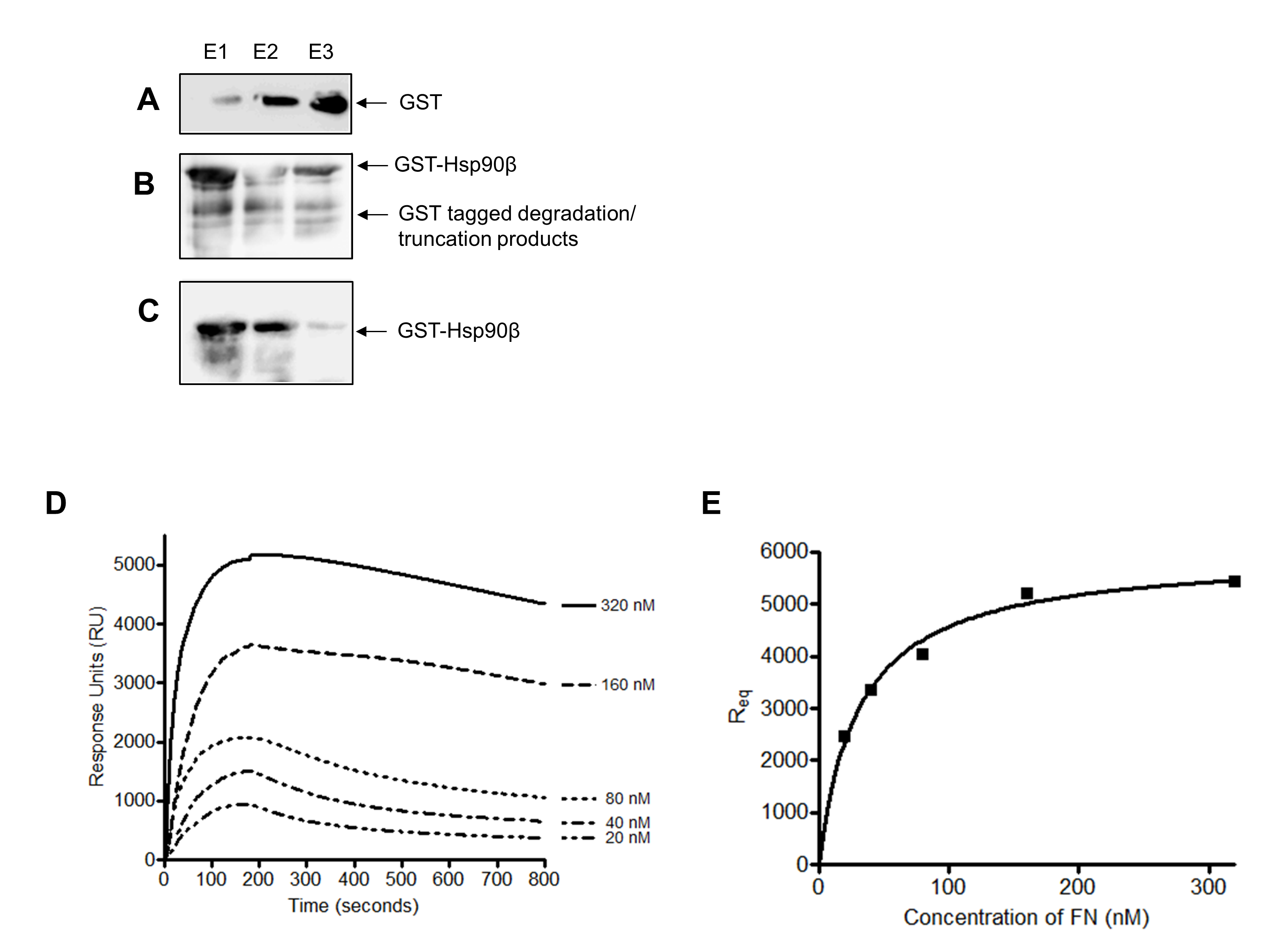

Supplement: Figure S1 — Hsp90β bound directly to FN in a dose dependant manner. Immunoblot of GST and GST-Hsp90β purifications used in the solid phase binding assay. Overproduction of GST-Hsp90β and GST occurred using the Escherichia coli BB1994 (MC4100 dnaK52 sidB1::Tc pDMI, 1::CmR KanR) strain as a host. Purification of GST-Hsp90β and GST occurred according to an adapted protocol of the batch purification of GST-tagged proteins in the Protino® Glutathione Agarose (GSH) 4B (745500, Macherey-Nagel, Germany) user manual. Immunoblots were probed using (A)+(B) anti GST and (C) anti Hsp90β antibodies. Letters above each immunoblot represent the collected elution fractions. Arrows show the purified GST, GST-Hsp90β and GST-tagged degradation/truncation products. (D) Representative sensorgrams of observed binding of FN (20, 40, 80, 160 and 320 nM) to immobilized Hsp90β (10 µg.ml−1). Hsp90β was immobilized onto the surface of a ProteOn™ GLM Sensor chip (#176-5012, Bio-Rad, US) and protein interaction was determined at 37°C. Sensorgrams are representative of quadruple experiments at varying FN concentrations (E) Saturation binding curve of FN-Hsp90β SPR binding data. Req values were plotted against concentration and fit with a non-linear curve (one site binding). An R2 value equal to 0.9800 was reported. (TIF) [file pone.0086842.s001.tif]

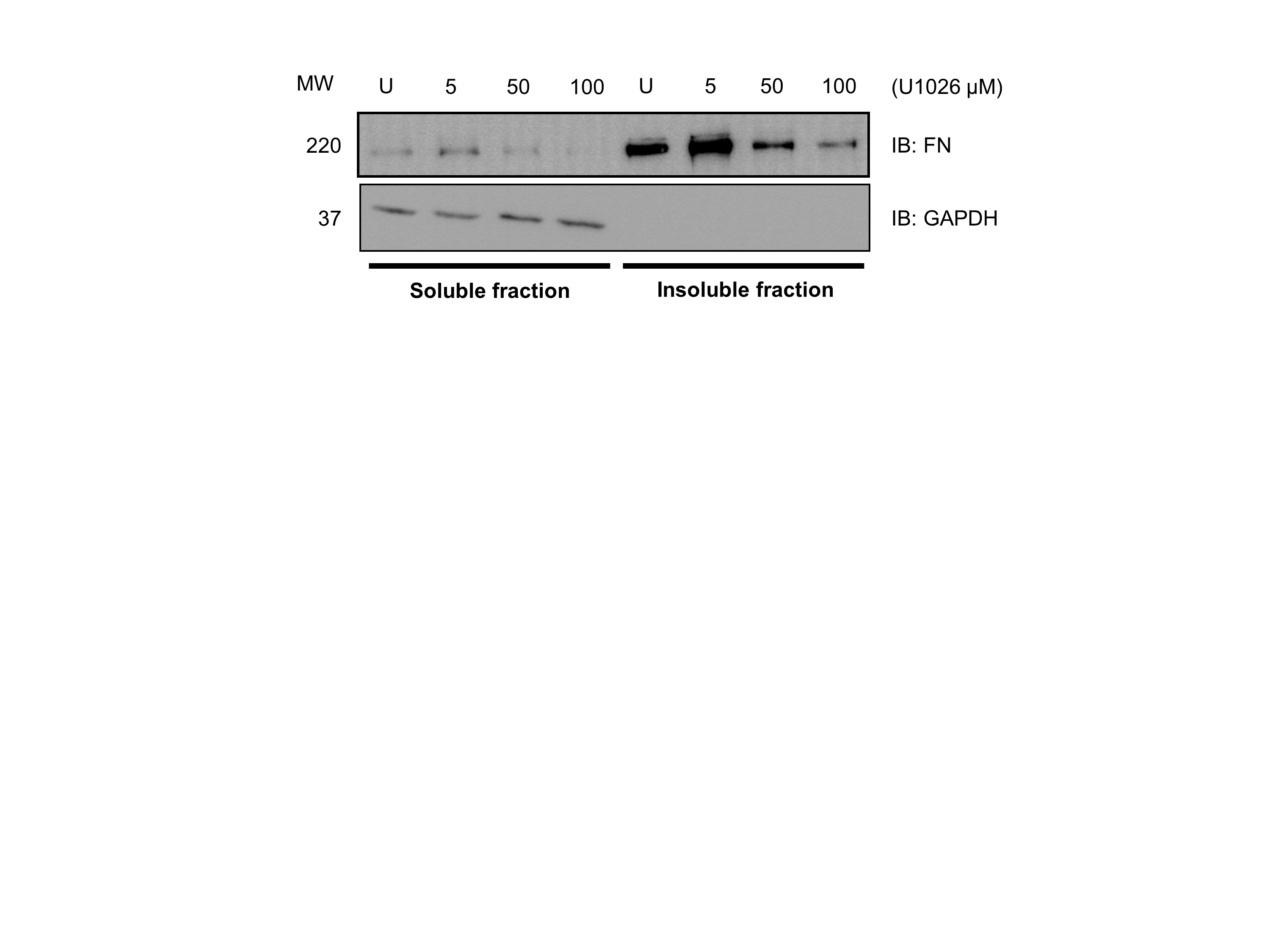

Supplement: Figure S2 — Validation of the deoxycholate (DOC) assay to quantitate FN matrix assembly in Hs578T breast cancer cells. Confluent Hs578T cells were incubated with increasing concentrations of the MEK1/2 inhibitor (U1026) for 16 hours at 37°C. Cells were lysed and DOC-soluble and DOC-insoluble FN fractions isolated using the described DOC assay and relative levels determined by immunoblotting. Analysis of GAPDH levels demonstrated no contamination between the soluble and insoluble fractions. (TIF) [file pone.0086842.s002.tif]

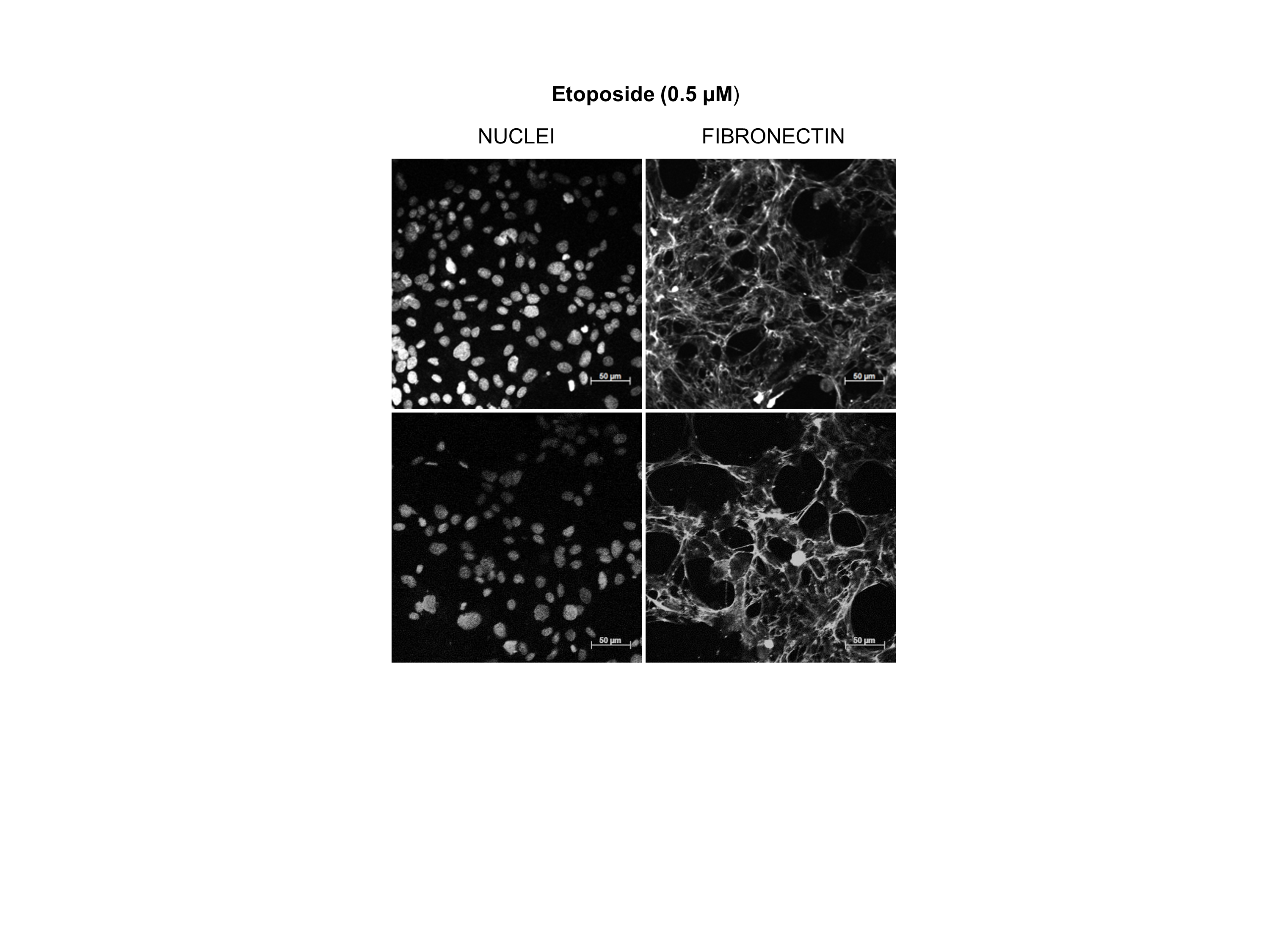

Supplement: Figure S3 — Etoposide has no effect on the extracellular FN matrix. Adherent Hs578T cells were treated with etoposide (0.5 µM or 1 µM) and the effect on the FN matrix examined by confocal microscopy. The IC50 value (0.96 µM) for etoposide has previously been determined in the Hs578T cell line [1]. Duplicate images of etoposide (0.5 µM) treatment are shown. Scale bars are equivalent to 50 µm. (TIF) [file pone.0086842.s003.tif]

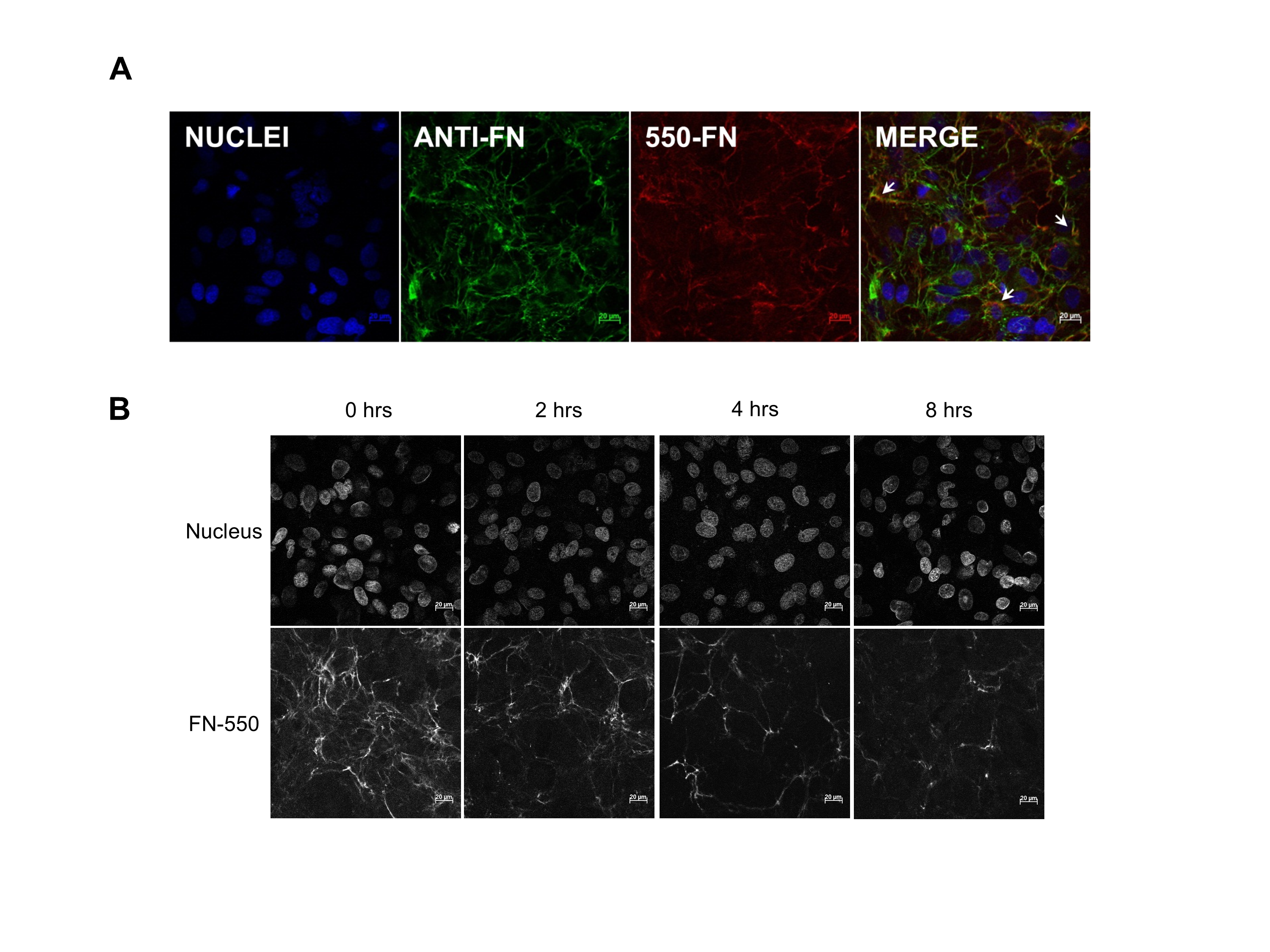

Supplement: Figure S4 — Analysis of fibronectin dynamics using exogenous fluorescent FN. (A) Cell imaging of exogenously added fluorescently labeled FN (FN-550). Hs578T cells were grown in phenol-red free media in sterile glass-bottomed microscopy culture dishes and allowed to undergo fibrillogenesis in media supplemented with FN-550 (50 nM). Fixed cells were stained using mouse anti human FN followed by donkey anti mouse DyLight® 488 fluorescent secondary antibodies. Images were captured using the Zeiss LSM 510 Meta confocal microscope. Scale bars are equivalent to 20 µm. White arrows show regions of incorporation of the exogenous FN-550 into the extracellular FN matrix. (B) Confluent Hs578T cells were allowed to undergo fibrillogenesis in media supplemented with FN-550 (50 nM). Following the confirmation of a fluorescent extracellular FN matrix, cells either remained untreated or were treated with novobiocin (NOV; 1 mM) for a period of 2, 4 or 8 hours. Cells were fixed and images captured using confocal microscopy. Three images were captured for each treatment in areas where similar cell numbers were observed. Scale bars are equivalent to 20 µm. Data are representative of three independent experiments with similar results. (TIF) [file pone.0086842.s004.tif]
